# d-Histidine inhibits *Streptococcus mutans* growth as a potential anti-caries agent

**DOI:** 10.1080/20002297.2025.2533174

**Published:** 2025-07-16

**Authors:** Liuchang Yang, Yuxing Chen, Yaqi Chi, Xiaolin Chen, Yiran Zhao, Mingrui Zhang, Xuemeng Wang, Yongliang Li, Jie Nie, Xiaoyan Wang

**Affiliations:** a Department of Cariology and Endodontology, Peking University School and Hospital of Stomatology & National Center for Stomatology & National Clinical Research Center for Oral Diseases & National Engineering Research Center of Oral Biomaterials and Digital Medical Devices & Beijing Key Laboratory of Digital Stomatology & NHC Key Laboratory of Digital Stomatology & NMPA Key Laboratory for Dental Materials, Beijing, China; b School of Stomatology, Hebei Medical University, Shijiazhuang, China

**Keywords:** Dental caries, *Streptococcus mutans*, D-amino acids, D-histidine, anti-caries agent

## Abstract

**Background:**

Dental caries is a bacterial-mediated infectious disease that affects the hard tissues of the tooth, with *Streptococcus mutans* being the primary cariogenic pathogen due to its robust biofilm-forming ability. Controlling biofilm formation is essential for caries prevention. Recent studies have indicated that D-amino acids, which are not incorporated into proteins, play regulatory roles in bacterial processes such as growth inhibition and biofilm dispersal. However, whether D-amino acids can inhibit the growth of *S. mutans* remains controversial. This study aimed to investigate the effects of D-amino acids on *S. mutans* growth and biofilm formation in vitro, as well as their anti-caries efficacy in a rat caries model.

**Materials and Methods:**

This study utilized *Streptococcus mutans* UA159 to screen 15 D-amino acids for growth inhibition, identifying D-histidine (D-His) as the most effective. Minimum inhibitory concentration, growth curves, biofilm assays, and transcriptomic analysis were performed in vitro. Anti-caries efficacy was evaluated in a rat model using Micro-CT and Keyes scoring.

**Results:**

D-His significantly inhibits the planktonic growth of *S. mutans* and delays biofilm formation, particularly in the early stages. Furthermore, RNA sequencing revealed 417 upregulated genes and 394 downregulated genes in D-His-treated *S. mutans*, with significant alterations in pathways related to carbohydrate utilization, protein biosynthesis, and transmembrane transport. Moreover, D-His exhibited effective caries prevention in an in vivo rat model.

**Conclusion:**

These findings suggest that D-His has potential as an anti-caries agent by targeting *S. mutans* growth and biofilm dynamics.

## Introduction

Dental caries remains one of the most prevalent chronic diseases worldwide, affecting billions of people across all age groups [1]. The disease is characterized by the demineralization of enamel and dentin of tooth, primarily driven by acid production from cariogenic bacteria within dental biofilms. Among these bacteria, *Streptococcus mutans* is considered a key pathogen due to its ability to form robust biofilms, produce acid from dietary carbohydrates, and tolerate acid environments [2]. Biofilms provide a protective niche for bacteria, making them resistant to mechanical removal and conventional antimicrobial agents [3]. Therefore, targeting biofilm formation is a promising strategy for caries prevention.

Amino acids are essential for bacterial growth and metabolism, with L-amino acids being the primary form used for protein synthesis. In contrast, D-amino acids, which are enantiomers of L-amino acids, are not involved in protein synthesis but are equally important for bacteria. D-amino acids are mainly produced by bacteria [4], and some D-amino acids have also been found in the oral cavity of human [5]. D-amino acids are integral to bacterial cell wall synthesis (D-Ala and D-Glu are incorporated in peptidoglycan) [6]. In addition, D-amino acids play important regulatory roles in bacterial physiology [7]. For example, D-amino acids can modulate bacterial growth, cell wall remodeling, and sporulation in various bacterial species [8].

Specifically, several studies have demonstrated that D-amino acids can inhibit biofilm formation or disperse existing biofilms [9]. For instance, Kolodkin-Gal et al. found that D-Leu, D-Met, D-Tyr, and D-Trp inhibit *Bacillus subtilis* biofilm formation and even disperse established biofilms. This anti-biofilm effect is mediated primarily through the replacement of D-alanine in peptidoglycan with non-canonical D-amino acids, which may disrupt the function of adhesion protein [10]. Similarly, Zhang et al. reported that D-Arg, D-Met, and D-His synergistically inhibit *Porphyromonas gingivalis* biofilm formation [11], while Zhang et al. showed that D-His enhances the efficacy of amikacin against *Pseudomonas aeruginosa* biofilms [12].

Up to now, whether D-amino acids can inhibit the growth of *S. mutans* remains controversial. A number of studies have explored combining some D-amino acids with antimicrobial agents to inhibit the biofilm formation of *S. mutans*. For example, Tong et al. demonstrated that D-Cys, D-Asp, and D-Glu, when combined with nisin, significantly enhance the inhibition of *S. mutans* biofilm formation [13]. Additionally, D-Cys has been shown to inhibit dual-species biofilms of *S. mutans* and *S. sanguinis* [14]. These findings highlight the potential of D-amino acids in caries prevention. However, these investigations have primarily focused on a limited subset of D-amino acids, and often in combination with other antimicrobial agents.

However, the sensitivity of different bacteria to D-amino acids varies significantly, and certain D-amino acids may act as unique community signaling molecules [15,16]. This variability underscores the need to study the effects of individual D-amino acids on *S. mutans* to develop targeted strategies for caries prevention.

**Figure 1.**
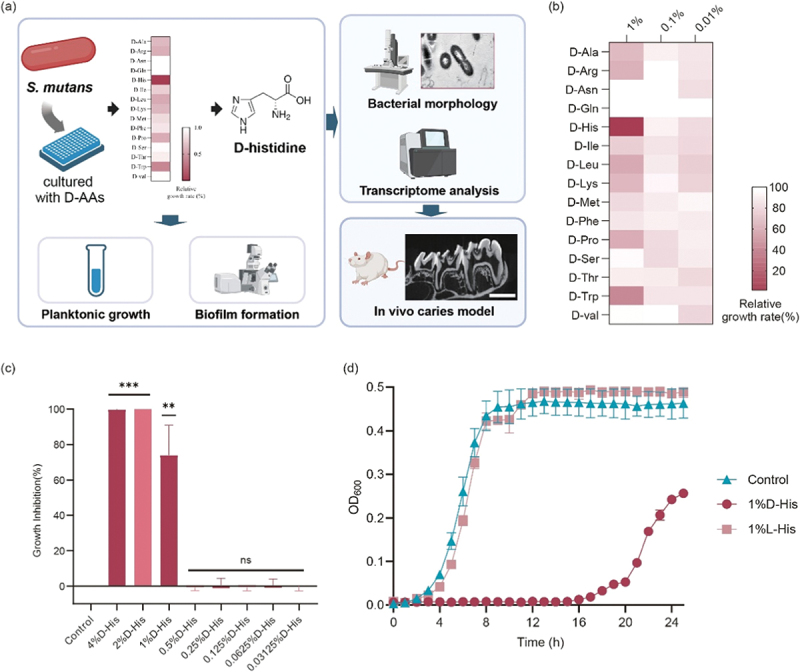
D-His delays growth of *S. mutans*. (a) Schematic illustration of the flow chart and experimental protocol of this study. (b) Growth inhibitory effects of different D-amino acids on *S. mutans* at different concentrations. (c) Determining the minimum inhibitory concentration (MIC) of D-His. The ordinate represents the growth inhibition rate of *S. mutans* at different concentrations of D-His during 24-hour planktonic growth. Data represent the means of three independent experiments, Student’s *t*-test were performed between each concentration group and the control group. ns: no significant difference; **: *p* < 0.01; ***: *p* < 0.001. (d) Growth curves of *S. mutans* under the effect of D-His and L-His.

This study aims to investigate the effects of D-amino acids on *S. mutans* growth, morphology, and biofilm formation, and evaluates the effect of D-His on *S. mutans* cariogenicity in a rat caries model. Furthermore, we try to explore the underlying mechanisms through transcriptomic analysis (Figure 1a).

## Materials and methods

### Bacterial strains and growth conditions

The *Streptococcus mutans* UA159 strain, a well-characterized cariogenic pathogen, was obtained from the Central Laboratory of Peking University School of Stomatology. The strain was stored at −80°C in Brain Heart Infusion (BHI) broth containing 20% glycerol. For experimental use, frozen stocks were streaked onto BHI agar plates and incubated at 37°C under 5% CO_2_ for 48 h. Single colonies were then inoculated into 10 mL of BHI broth and grown overnight. The optical density at 600 nm (OD_600_) of the overnight culture was measured, and the bacterial concentration was adjusted to 10^9^ CFU/mL with sterile BHI broth. This suspension served as the working bacterial inoculum for all subsequent experiments.

### Preparation of D-amino acid solutions

D-alanine (D-Ala) and D-arginine (D-Arg) were purchased from Sigma-Aldrich (St. Louis, MO, USA). Other D-amino acids, including D-asparagine (D-Asn), D-glutamine (D-Gln), D-histidine (D-His), D-isoleucine (D-Ile), D-leucine (D-Leu), D-lysine (D-Lys), D-methionine (D-Met), D-phenylalanine (D-Phe), D-proline (D-Pro), D-serine (D-Ser), D-threonine (D-Thr), D-tryptophan (D-Trp) and D-valine (D-Val), were purchased from Aladdin (Shanghai, China). All compounds met molecular biology purity standards (> 98% by HPLC). Each D-amino acid was dissolved in sterile BHI broth to final concentrations ranging from 0.01% to 1% (w/v). The pH of the solutions was adjusted to 7.4, and the solutions were filter-sterilized using a 0.22 μm pore-size membrane (Millipore, Billerica, MA, USA). Solutions were prepared fresh on the day of the experiment to avoid degradation.

### Screening of D-amino acid sensitivity

To assess the sensitivity of *S. mutans* to D-amino acids, 100 μL of the working bacterial inoculum (10^9^ CFU/mL) was added to 10 mL of BHI broth containing 0.01%–1% (w/v) of each D-amino acid. Cultures were incubated at 37°C under 5% CO_2_ for 12 h. Bacterial growth was monitored by measuring OD_600_ using a microplate reader (SpectraMax190, Molecular, USA). Relative growth was calculated as the ratio of OD_600_ in the presence of D-amino acids to that of the control (BHI broth without D-amino acids). Experiments were performed in triplicate.

### Determination of minimum inhibitory concentration (MIC)

The MIC of D-His against *S. mutans* was determined using a broth microdilution method. Serial two-fold dilutions of D-His were prepared in BHI broth, with final concentrations ranging from 0.03125% to 4% (w/v). A 100 μL aliquot of the bacterial inoculum (10^9^ CFU/mL) was added to each well of a 96-well microtiter plate containing 100 μL of the D-His solutions. The plate was incubated at 37°C under 5% CO_2_ for 24 h. The MIC was defined as the lowest concentration of D-His that resulted in no visible bacterial growth (OD_600_ change <0.01). Each concentration was performed in triplicate.

### Bacterial growth curve analysis

The effect of D-His on *S. mutans* growth kinetics was assessed by monitoring OD_600_ over time. Overnight cultures of *S. mutans* were diluted 1:100 in BHI broth containing 1% D-His or 1% L-His. Aliquots of 100 μL were transferred to a 96-well microtiter plate, and OD_600_ was measured every hour for 24 h using a microplate reader (SpectraMax190, Molecular, USA). Growth curves were plotted, and the lag-phase duration, exponential growth rate, and maximum OD_600_ were calculated. Experiments were performed in triplicate.

### Live cell microscopy imaging

1.5% (w/v) low-melting agarose (Takara Bio, Japan) was dissolved in BHI broth supplemented with 1% (w/v) D-His and heated to ensure complete dissolution. Aliquots of 50 μL molten agarose were pipetted onto pre-cleaned glass slides and allowed to polymerize at room temperature. Following polymerization, 1 μL of *S. mutans* overnight culture (10^9^ CFU/mL) was inoculated onto the gel surface. After air-drying, the samples were sealed with coverslips and gently pressed to eliminate air bubbles. Time-lapse imaging was conducted at one-hour intervals using an N-STORM microscope system (Nikon, Japan) equipped with a 100× oil immersion objective. Raw image stacks were processed using NIS-Elements (Nikon, Japan) and analyzed with ImageJ.

### Biofilm formation assay

Biofilms were grown in BHIS medium (BHI supplemented with 0.75% sucrose) to mimic the cariogenic environment. *S. mutans* was inoculated into BHIS with or without 1% D-His to a final concentration of 10^6^ CFU/mL. Aliquots of 200 μL were transferred to 96-well microtiter plates and incubated at 37°C under 5% CO_2_ for 6, 12, 18, and 24 h. After incubation, biofilms were washed three times with PBS to remove non-adherent cells, then fixed with methanol for 1 min, and stained with 0.5% crystal violet for 30 min. Excess stains were removed by washing with distilled water, and the stained biofilms were solubilized with 200 μL of methanol. Biofilm biomass was quantified by measuring OD_570_ using a microplate reader (SpectraMax190, Molecular, USA). Experiments were performed in triplicate.

### Confocal laser scanning microscopy (CLSM)

For three-dimensional analysis of biofilms, *S. mutans* was cultured in BHIS with or without 1% D-His in 35 mm glass-bottom dishes (Cellvis, USA). After 12 or 24 h, biofilms were washed with PBS and stained using the LIVE/DEAD BacLight Bacterial Viability Kit (Thermo Fisher Scientific, USA). SYTO 9 (0.5 μmol/L) and propidium iodide (3 μmol/L) were used to stain live and dead cells, respectively. Biofilms were imaged using a confocal laser scanning microscope (Leica SP8, Germany) with a 25× oil immersion objective. Z-stacks were acquired at 2 μm intervals, and biofilm cell biomass, average thickness, roughness coefficient and surface-to-biovolume ratio were analyzed using LAS X software (Leica, Germany) and Comstat2 software (www.comstat.dk) [17]. Experiments were performed in triplicate.

### Transmission electron microscopy (TEM)

To examine the morphological changes induced by D-His, *S. mutans* cells were cultured in BHI broth with or without 1% D-His for 7 h. Bacterial cells were harvested by centrifugation at 3,000 × g for 15 min and fixed with 2.5% glutaraldehyde in PBS (pH 7.4) for 2 h at room temperature. After washing with phosphate-buffered saline (PBS), the cells were post-fixed with 1% osmium tetroxide for 2 h, dehydrated in a graded ethanol series (30%, 50%, 70%, 80%, 85%, 90%, and 100%), and embedded in epoxy resin. Ultrathin sections (70 nm) were cut using an ultramicrotome (Leica UC7, Germany), stained with uranyl acetate and lead citrate, and examined using a transmission electron microscope (TECNAI G 20 TWIN, FEI, USA). Cell wall thickness was measured using ImageJ software, and at least 50 cells were analyzed per group.

### RNA sequencing and transcriptomic analysis

Total RNA was extracted from *S. mutans* cells cultured with or without 1% D-His for 7 h using the RNAprep Pure Cell/Bacterial Kit (Tiangen, China). RNA quality was assessed using an Agilent 2100 Bioanalyzer (Agilent Technologies, USA). RNA sequencing was performed on an Illumina NovaSeq PE150 platform (Illumina, USA). Raw reads were filtered and aligned to the *S. mutans* UA159 reference genome (NCBI accession: NC_004350.2) using HISAT2. Differential gene expression analysis was performed using DESeq2, with genes showing |log2FC| ≥1 and *padj* <0.05 considered significantly differentially expressed. Gene Ontology (GO) and Kyoto Encyclopedia of Genes and Genomes (KEGG) pathway analyses were conducted using the clusterProfiler R package.

### Quantitative PCR (qPCR) assays

The RNA extraction was the same as transcriptome sequencing. Reverse transcription was performed using the Hifair one-step RT-gDNA SuperMix (Yeason, China). Real-time PCR was performed for the quantification of *pbp2b*, *ddl*, *murE*, *pbp2x*, *mraY*, and *murA* mRNA expression of the *S. mutans* in D-His groups, with 16S rRNA as an internal control. Primers for each gene are listed in Table 1. Real-time PCR amplification was performed on QuantStudio 3 (Thermo Fisher Scientific, USA). The reaction mixture (20 μL) contained 1 × SYBR green PCR Master Mix (Yeason, China), template cDNA, and forward and reverse primers (0.2 μmol/L). Threshold cycle values (CT) were determined, and the relative gene expression was calculated according to the 2^−ΔΔCT^ method. Experiments were performed in triplicate.

**Table 1.** Sequences of qPCR primers used in this study.

Gene	Forward primer	Reverse primer
*16S rRNA*	ATTGTTGCTCGGGCTCTTCCAG	ATGCGGCTTGTCAGGAGTAACC
*pbp2b*	TGCTCTTGGAGCGGTTACTG	TAGGGCTTGAACCCGCAAAT
*ddl*	CTTTCCGAACTTTGGGCTGC	AAAGCCGGGCATAGTGTTGA
*murE*	CCGTCCGGCCATGATTTCTA	ACACGGCCAACTAGGTAAGC
*pbp2x*	TGGACTATGTTGTGGGCGAC	ACAGAGGACGCCCCTTTAGA
*mraY*	GGGCCACCTTGGCTATTCTT	GACCGCCAATAAGCTGCAAG
*murA*	ATGCACGGTACTGACCACAG	TCTTCAGTGACTTGGACGCC

### Anti-caries effect in a rat caries model

The protocol of animal experiment was derived from previous studies [18] with some modifications and approved by the School of Stomatology, Peking University Animal Ethics Committee (BDKQ-202502070072).

Seventeen-day-old SPF-grade male Sprague-Dawley (SD) rats were used to establish a dental caries model. Throughout the experiment, the rats of both groups were fed a cariogenic diet (Diet 2000) [18] and 10% sucrose water, with their body weights measured regularly. During the first 3 days (days 1–3), the rats were provided with 0.05% (w/v) streptomycin in their drinking water. From day 4 to 8, the rats’ oral cavities were inoculated with *S. mutans* twice a day. The suspension of *S. mutans* (10^9^ CFU/mL) was taken with a sterile cotton swab and smeared, respectively, on the left and right molar regions 20 times, then 50 μL of bacterial suspension was instilled into the oral cavity of the rat. After inoculation, no food or water was allowed for 30 min. Before the first inoculation on the morning of day 4 and before the start of treatment on the morning of day 9, oral samples were collected and cultured on mitis-salivarius-bacitracin agar (MSA) plates to confirm the successful infection.

Subsequently, the rats were randomly divided into two groups (*n* = 3). From day 9 to 36, the molars of each rat were administered 50 μL of the treatment according to the group assignment locally using a sterile cotton swab, applied for 30 s, twice daily for 4 weeks. The experimental group received 1% D-His treatment, while the control group received distilled water treatment.

On day 37, the rats were euthanized using CO_2_ gas. The mandibles were collected, fixed in 4% paraformaldehyde, and scanned using Micro-CT (Skyscan 1276, Bruker, USA). After scanning, the specimens were stained with 0.4% ammonium purpurate for 24 h, and the rat molars were sectioned in the mesiodistal direction. Caries lesions were observed under a stereomicroscope (SZ61, Olympus, Japan) and scored using the Modified Keyes caries scoring method [19].

### Statistical analysis

All experiments were performed in triplicate, and the data were expressed as mean ± SD. Statistical analysis was performed using SPSS 27.0 (IBM, USA) and GraphPad Prism 10.1 (GraphPad Software, USA). Student’s *t*-test was used for comparisons between two groups, and one-way ANOVA followed by Tukey’s post hoc test was used for multiple comparisons. The Mann-Whitney U-test was used to analyze the Keyes’ scores between two groups in the rat caries model. *P*-value <0.05 was considered statistically significant.

## Results

### *D-His delays growth of* S. mutans

To investigate the effect of D-amino acids on the viability of *S. mutans*, we screened 15 available D-amino acids and examined their effects on *S. mutans* growth at various concentrations (1% to 0.01%, w/v). As shown in Figure 1b, *S. mutans* exhibited differential sensitivity to D-amino acids. Among the tested amino acids, 1% D-His exhibited the strongest inhibitory effect, reducing relative growth to less than 20% of the control. Moderate inhibition (with relative growth ranging from 0.2 to 0.8) was observed for D-Arg, D-Ile, D-Leu, D-Lys, D-Met, D-Pro, and D-Trp at 1%. Mild inhibition (relative growth >0.8) was noted for D-Phe, D-Ser, D-Thr, and D-Val at 1%. The inhibitory effect, however, diminished as the concentration decreased. Notably, D-Asn and D-Gln had no significant impact on *S. mutans* growth in this study. These results suggest that some D-amino acids, especially D-His, possess growth-inhibitory effects on *S. mutans.*

Next, the minimum inhibitory concentration (MIC) of D-His for *S. mutans* was determined to be 2% (w/v) (Figure 1c). Based on this MIC value, a sub-inhibitory concentration of 1% D-His was selected to investigate its effect on growth modulation without completely suppressing bacterial growth. The growth curve (Figure 1d) showed that 1% D-His significantly prolonged the lag phase of *S. mutans* and reduced the growth rate during the exponential phase. The final optical density (OD_600_) of the D-His-treated culture was significantly lower than that of the control (*p* < 0.05). In contrast, 1% L-His did not exhibit any inhibitory effect, underscoring the unique activity of the D-enantiomer.

### *D-His delays biofilm formation of* S. mutans

**Figure 2.**
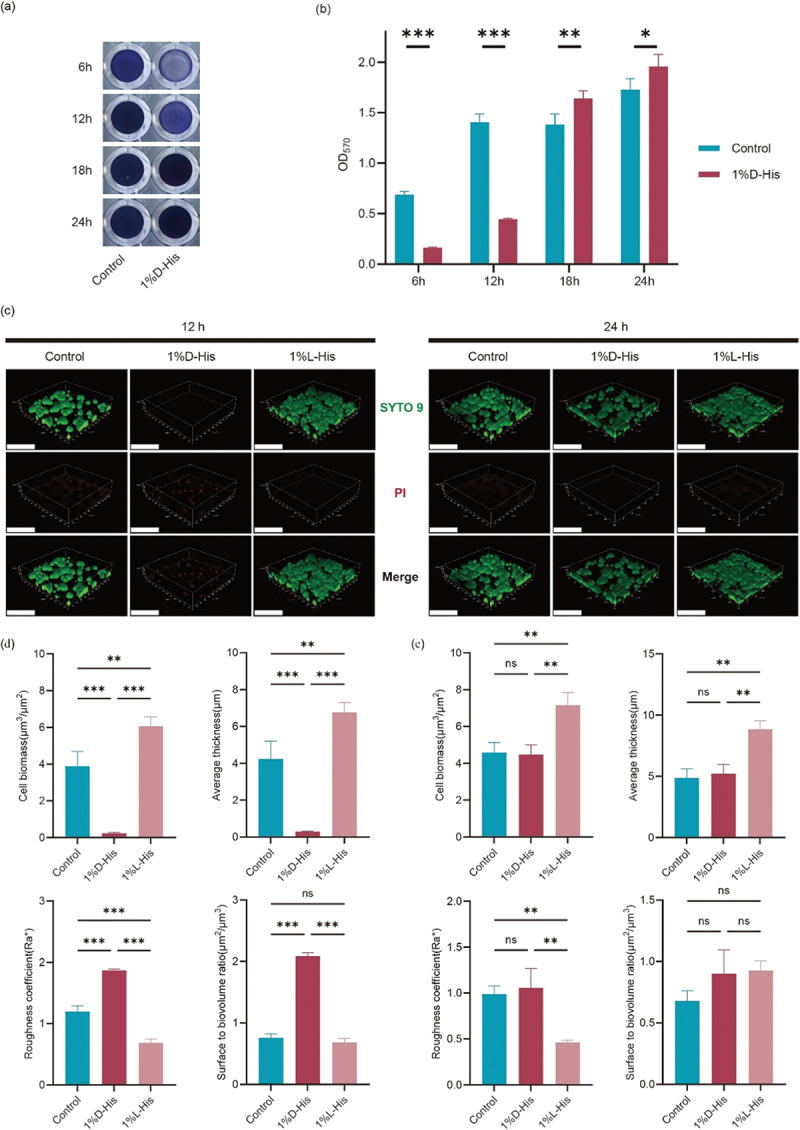
D-His delays biofilm formation of *S. mutans*. (a) Crystal violet staining results of biofilms in the D-His group and control group at different time points. (b) Quantitative analysis of the biofilms shown in panel (a) by absorbance (OD₅₇₀). Data represented the means of three independent experiments, student’s *t*-test were performed for pairwise comparisons between the two groups at each time point. *: *P* < 0.05; **: *P* < 0.01; ***: *P* < 0.001. (c) Representative confocal laser scanning microscopy (CLSM) images of *S. mutans* biofilms treated with D-His. Left panels: 12 h biofilm; right panels: 24 h biofilm. (d) Measurement of biofilm parameters treated with D-His for 12 h from CLSM analysis (corresponding to left panels in c): cell biomass, average thickness, roughness coefficient and surface to biovolume ratio. (e) Measurement of biofilm parameters treated with D-His for 24 h from CLSM analysis (corresponding to right panels in c). For (d) and (e), data represented the means of three independent experiments, one-way ANOVA was performed for intergroup comparisons. **: *P* < 0.01; ***: *P* < 0.001.

The inhibitory effects of D-His on *S. mutans* biofilm development were systematically evaluated through crystal violet quantification and confocal laser scanning microscopy (CLSM). Quantitative analysis revealed a significant suppression of biofilm biomass in the D-His-treated group at 12 h (OD_570_: 0.44 ± 0.01 vs. control 1.40 ± 0.08, *p* < 0.001). However, this inhibitory effect was not sustained, as biofilm accumulation in the D-His group not only recovered but surpassed control levels by 24 h (OD_570_: 1.96 ± 0.06 vs. 1.73 ± 0.11, *p* < 0.05) (Figure 2a,b).

CLSM three-dimensional reconstructions revealed distinct temporal dynamics. At 12 h, D-His treatment induced a 90% reduction in biofilm cell biomass (0.24 ± 0.04 vs. control 3.89 ± 0.78 μm^3^/μm^2^, *p* < 0.001), accompanied by reduced vertical growth (average thickness: 0.29 ± 0.03 vs. 4.22 ± 0.98 μm, *p* < 0.001), increased surface roughness (1.87 ± 0.02 vs. 1.20 ± 0.09 Ra*, *p* < 0.001), and enhanced porosity (surface-to-biovolume ratio: 2.09 ± 0.05 vs. 0.76 ± 0.06 μm^2^/μm^3^, *p* < 0.001). By 24 h, all structural parameters (cell biomass, thickness, roughness, and surface-to-biovolume ratio) had returned to control levels, indicating complete functional recovery of biofilm-forming capacity. In contrast, 1% L-His consistently promoted hyper-biofilm formation, with increased biomass deposition and thickened architecture throughout the experimental period (Figure 2c–e).

### *Cell morphology and transcript analysis of* S. mutans *treated with D-His*

**Figure 3.**
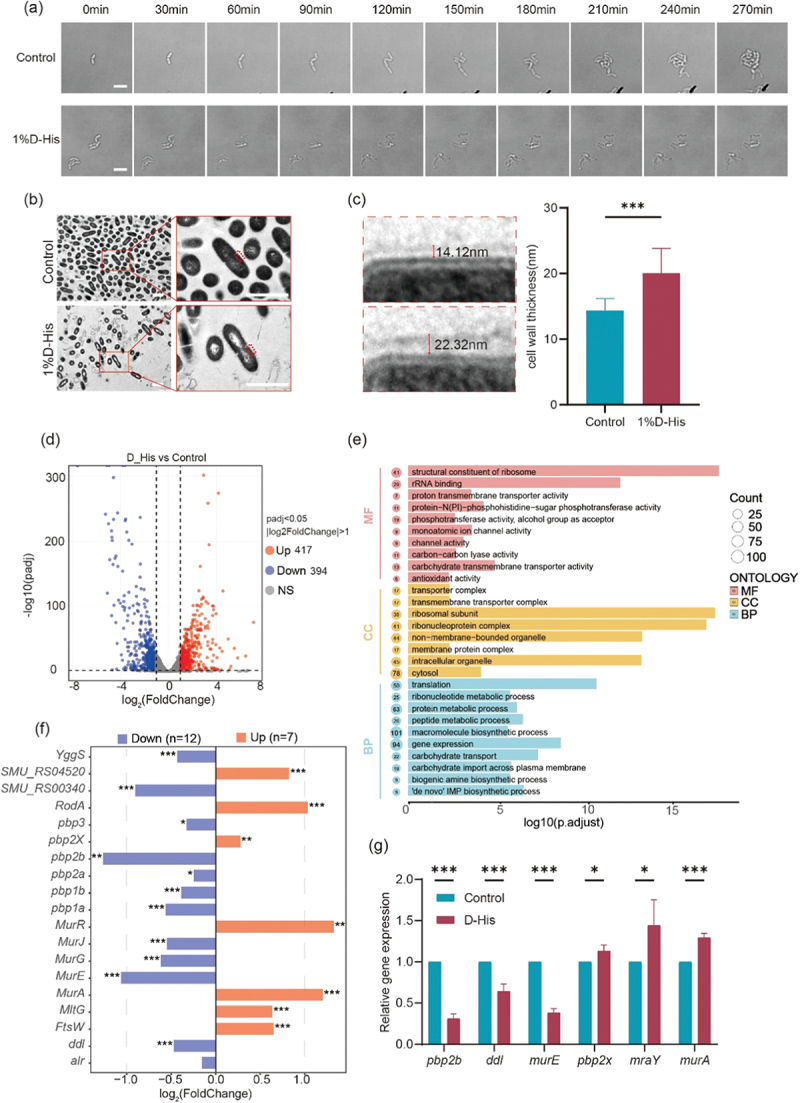
Cell morphology and transcript analysis of *S. mutans* treated with D-His. (a) The representative images of the proliferative morphology of *S. mutans* treated with or without D-His (scale bar = 2 μm). (b) The representative images of morphological changes of *S. mutans* treated with D-His by transmission electron microscopy. Transmission electron micrographs of *S. mutans* in the blank control group and 1% D-His group (scale bar = 1 μm). (c) Cell wall thickness measurements of *S. mutans* in the blank control group and 1% D-His group (*n* = 60 cells). Student’s *t*-test was performed for comparison between the two groups, with *p* < 0.001. (d) RNA-Seq results analysis and volcano plot analysis of differentially expressed genes (DEGs). (e) Geno ontology (GO) pathway enrichment analysis of the DEGs. (f) Expression of genes associated with peptidoglycan synthesis. (g) Quantitative qPCR results of gene expression in *S. mutans* treated with D-His. Student’s *t*-test was used for intergroup comparisons. *: *p* < 0.05; ***: *p* < 0.001.

To investigate the inhibitory mechanism of D-His on *S. mutans*, we first examined changes in cell morphology following D-His treatment. Live-cell microscopy images revealed that, in the presence of 1% D-His, *S. mutans* experienced significant growth inhibition, requiring 270 min to complete one division cycle, whereas the control strain completed a division cycle in just 60 min (Figure 3a). Although the growth rate was affected by 1% D-His, the cell length and width remained unchanged. This finding was further confirmed by transmission electron microscopy (TEM) analysis. Notably, TEM images showed that 1% D-His treatment led to an increased cell wall thickness (*p* < 0.001). The average cell wall thickness in the D-His-treated group was 20.07 ± 3.78 nm, compared to 14.36 ± 1.84 nm in the control group (Figure 3b,c).

Next, we conducted transcript sequencing (RNA-Seq) to further explore the mechanism by which D-His inhibits the growth of *S. mutans*. Since 1% D-His significantly inhibited *S. mutans* planktonic growth without exerting bactericidal effects, this concentration was selected to investigate transcriptional changes under D-His treatment. Differential expression analysis (*padj* <0.05, |log2FoldChange| >1) identified 417 upregulated and 394 downregulated genes in the D-His-treated group compared to the control (Figure 3d).

Gene Ontology (GO) enrichment analysis revealed distinct functional patterns across molecular hierarchies. At the molecular function level, differentially expressed genes (DEGs) were primarily associated with ribosomal structural constituents, rRNA binding, phosphotransferase activity, and carbohydrate transmembrane transport. Biological process analysis highlighted enrichments in carbohydrate transport, macromolecule biosynthesis, gene expression, protein translation, and metabolic processes. Cellular component analysis revealed clusters related to the ribosomal complex and transmembrane transport complex (Figure 3e).

Among the differentially expressed genes (Table S1), genes involved in bacterial division (*ftsZ, ftsA*), carbohydrate transport and metabolism (*celB, lacF, ptsP, pfkA, treP, lacA, lacB, gtfD, glmM, treC*), and environmental adaptation (*lrgA, lrgB*) were significantly downregulated. In contrast, genes encoding ribosomal proteins (*rpsS, rpsQ, rpsI, rplK, rplS, rplM, rplQ, rpsK, rpsT*), arginine biosynthesis enzymes (*arcC, argH, argF, argC, argJ, gdhA*), and the key biofilm matrix synthase gene, *gtfB*, were upregulated. Additionally, genes related to the CiaRH system (*ciaR, ciaH*) and the VicRK system (*vicK*) showed significant upregulation.

In addition to the downregulation of division-associated genes (*ftsZ*, *ftsA*), which was consistent with the observed phenotypes (slower bacteria division). Notably, we also found abnormal expression of genes involved in peptidoglycan synthesis, which is consistent with the observed phenotype of abnormal cell wall structure. The expression of penicillin-binding proteins (*pbp1a, pbp1b, pbp2a, pbp2b*), as well as *murE, murG*, and *murJ*, was downregulated, while the expression of *murR, murA, ftsW*, and *rodA* was upregulated (Figure 3f). To further validate the RNA-seq results, we performed qPCR analysis on *pbp2b, ddl, murE, pbp2x, mraY*, and *murA*. The expression trends observed by qPCR were consistent with those obtained from RNA-seq (Figure 3g). These findings suggest a functional shift in carbohydrate utilization, protein biosynthesis, and transmembrane transport in response to D-His treatment.

### D-His prevents caries in rat caries model

**Figure 4.**
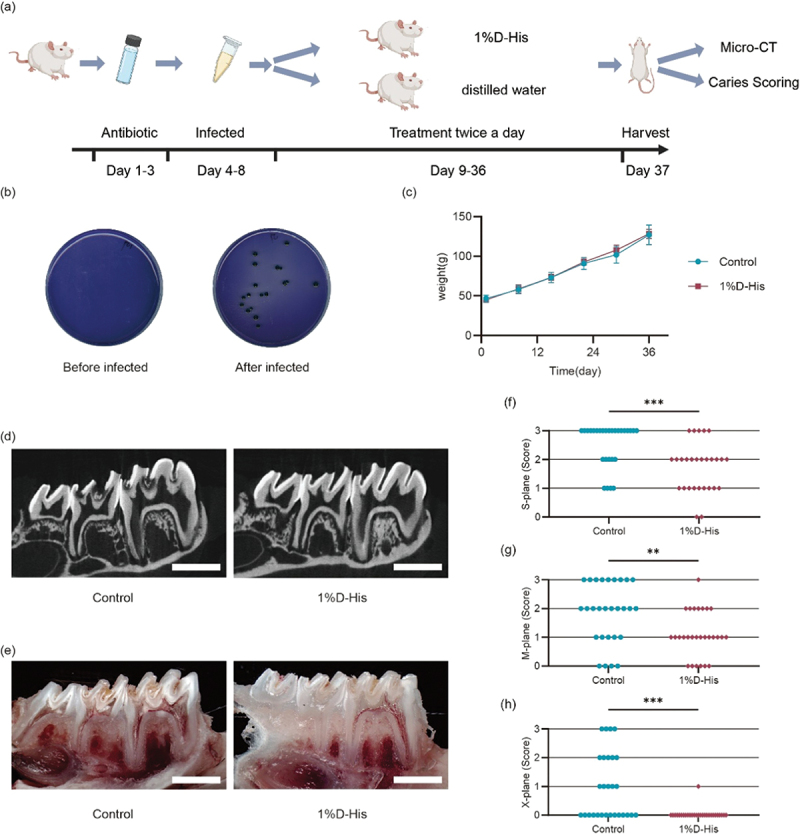
D-His prevent caries in rat caries model. (a) Overview of the procedure for establishing a rat caries model. (b) Colonies of *S. mutans* strain were successfully detected after oral inoculation. (c) Body weight changes in rats. (d) Micro-CT scans of rat mandibles (scale bar = 2 mm). (e) Longitudinal section stained images of rat mandibles (scale bar = 2 mm). (f-g) modified Keyes caries scores of rat mandibular teeth (S-plane, M-plane, X-plane). Mann–Whitney U test was performed for intergroup comparisons. **: *p* < 0.01; ***: *p* < 0.001.

To investigate the *in vivo* anti-caries efficacy of D-His, a rat model of dental caries was established (Figure 4a). Following antibiotic pretreatment, no streptococci were detected in the oral cavity prior to *S. mutans* inoculation. After five consecutive days of *S. mutans* inoculation, the bacteria were successfully detected in all rats through MSA plate culture (Figure 4b). Over the course of 4 weeks of treatment, both the control and D-His groups showed normal weight gain, with no significant differences between the groups (Figure 4c). Micro-CT analysis of enamel and dentin caries, with representative images shown in Figure 4d, revealed a significantly reduced depth of dentin demineralization in the D-His group compared to the control group. Furthermore, no enamel breakdown was observed at the fissure sites in the D-His group. Ammonium purpurate staining and sectioning showed a greater prevalence of moderate and deep carious lesions in the control group, while the D-His group predominantly exhibited superficial lesions (Figure 4e). Modified Keyes scoring revealed significantly lower S-plane, M-plane, and X-plane scores in the D-His-treated rats compared to the control group (Figure 4f–h). These results demonstrate the promising anti-caries potential of D-His *in vivo*.

## Discussion

Dental caries remains a significant global health challenge, with *S. mutans* playing a central role in its pathogenesis due to its ability to form robust biofilms and produce acid. This study demonstrates that D-His, a D-amino acid, effectively inhibits *S. mutans* planktonic growth and delays biofilm formation, highlighting its potential as an anti-caries agent.

The inhibitory effects of D-His on *S. mutans* share similarities and differences with other D-amino acids. For instance, D-Cys has been shown to inhibit dual-species biofilms of *S. mutans* and *S. sanguinis* by disrupting extracellular polysaccharide (EPS) synthesis [14], but its activity requires co-treatment with other D-amino acids to achieve significant biofilm suppression [13]. In contrast, D-His alone exhibits potent anti-biofilm effects at sub-MIC concentrations (1%), suggesting their anti-biofilm effects may operate through different ways. Similarly, D-Arg and D-Met synergistically inhibit the growth of *P. gingivalis* [11], yet they show weaker activity against *S. mutans* compared to D-His in the present study. These variations may stem from species-specific differences in D-amino acid uptake or metabolic integration. Notably, the inhibitory effect of D-Trp on the growth of *S. mutans* was not as strong as that of *Bacillus subtilis*, in which D-Trp could even effectively disperse the *Bacillus subtilis* biofilm [15]. These findings reinforce the notion that the anti-biofilm efficacy of D-amino acids is highly context-dependent, influenced by bacterial species and environmental factors. The same concentration of L-His did not affect the planktonic growth of *S. mutans*, which further indicated that there were different effects of chiral molecules.

The delayed biofilm formation observed in this study is particularly noteworthy. D-His significantly reduced biofilm biomass and viability at 12 h. The eventual recovery of biofilm formation by 24 h suggests that *S. mutans* can adapt to D-His stress (Figure 2a,c). One reason for this adaptation is that *S. mutans* could employ compensatory mechanisms to promote biofilm formation, this hypothesis is consistent with the upregulation of *gtfB* in RNA-seq, a key gene for extracellular polysaccharide synthesis and biofilm formation [20,21]. Similar compensatory responses have been reported in *S. mutans* exposed to sub-MIC of antimicrobial agents [22,23]. However, as we have not observed that D-His could disperse the established biofilm of *S. mutans* (data not shown in results). Therefore, another plausible explanation is that the biofilm reduction might be caused by the delayed growth of *S. mutans* under D-His stress. At 12 h, the D-His-treated group not only exhibited reduced biofilm biomass but also showed increased surface roughness and porosity (surface-to-biovolume ratio), which may facilitate deeper penetration of antimicrobial agents into the biofilm during early stages, thereby enhancing biofilm eradication [24]. If combined with other antimicrobial agents, this characteristic could further improve the efficacy of biofilm suppression.

Among all D-amino acids, D-Ala and D-Glu are the intrinsic components of peptidoglycan side chains, and are involved in cell wall synthesis and cross-linking [25]. D-amino acids could affect the composition, quantity, and strength of peptidoglycan, by being incorporated into the peptidoglycan strucutre and by regulating the synthesis and modification enzymes [8,26]. In this study, we found that D-His treatment resulted in an increased cell wall thickness (Figure 3b). Two reasons might explain this phenotype, one is that D-His may disrupt the cell wall synthesis leading to a looser cell wall structure, which is consistent with previous studies. But we cannot exclude the other one is that *S. mutans* may enhance cell wall synthesis as a protective mechanism [27].

Consistent with the change of cell wall thickness, there was a significant downregulation of gene expression of the penicillin-binding protein PBP2b, a transpeptidase that catalyzes cross-linking between stem peptides during cell wall synthesis. At the same time, UDP-N-acetylglucosamine enolpyruvyl transferase (MurA) is the initial step in biosynthesis cell wall synthesis [28]. Here, we observed a downregulation of *pbp2b*, while *murA* expression increased (Figure 3f) [25], we hypothesized that D-His might disrupt peptidoglycan crosslinking, perhaps through competitive inhibition of D-Ala-dependent transpeptidases [29]. This defect could trigger compensatory cell wall remodeling, leading to thickened yet structurally weakened envelopes.

The downregulation of *ftsZ* and *ftsA* correlates with impaired cell division [30], as evidenced by the prolonged lag phase. Notably, the downregulation of PTS transporters (*ptsP*) and glycolytic enzymes (*pfkA*) indicates a metabolic bottleneck that restricts carbohydrate utilization [31], potentially limiting the energy available for initial biofilm formation. The dramatic upregulation of *gtfB* correlates with biofilm recovery at 24 h, likely through the compensatory synthesis of water-insoluble glucans that reinforce the biofilm architecture [20]. This biphasic response underscores the need for combinatorial strategies to exploit the transient window (12 h) before *S. mutans* activates stress-adaptive pathways. And the upregulation of ribosomal genes may be a compensatory response due to disturbed protein synthesis [32,33], which may be caused by the interference of D-His [33]. Transcriptomic results indicate that under the stimulation of D-His, the expression of various substance transport proteins on the membrane and the defense mechanisms against adverse environments are significantly inhibited, particularly the transport of carbohydrates into the cell. Concurrently, the expression of genes related to intracellular gene expression and protein translation is markedly upregulated (Figure 3e). We speculate that the stimulation of D-His induces *S. mutans* to enter a persister-like state. This hypothesis is consistent with the phenotypic experimental observations that the inhibitory effect of D-his on the growth of *S. mutans* weakens over time.

Although we have found that D-His cannot kill or permanently inhibit the growth of *S. mutans*, our animal experiment demonstrated that D-His suppressed the cariogenic ability of *S. mutans in vivo* (Figure 4). This suggests the potential application of D-His as an anti-caries agent. However, due to the lack of sufficient evidence, it is not possible to conclusively determine whether D-His is specifically effective against *S. mutans* or a broad-spectrum antimicrobial agent. Meanwhile, the BHI medium may contain small amounts of D-amino acids from brain extract [34,35]. The effect of these amino acids on the growth of *S. mutans* cannot be completely excluded and may interfere with the determination of the effect concentration. Future studies utilizing a chemically defined medium will make the results more solid. Moreover, given that the dental caries was caused by multiple species, this study only explored the role of D-His on *S. mutans*, and further exploration of the effect on other cariogenic bacteria is needed.

## Data Availability

The data that support the findings of this study are available from the corresponding author upon reasonable request.
